# Three-Dimensional Variation of the Human Hard Palate Across Populations: A Geometric Morphometrics Study

**DOI:** 10.3390/dj14050258

**Published:** 2026-04-29

**Authors:** Thao Liang Chiam, Angela Gurr, Toby Hughes, Lyle Palmer, Denice Higgins

**Affiliations:** 1 School of Dentistry, College of Health, Adelaide University, Adelaide, SA 5005, Australia; angela.gurr@adelaide.edu.au (A.G.); toby.hughes@adelaide.edu.au (T.H.); denice.higgins@adelaide.edu.au (D.H.); 2 Faculty of Dentistry, The National University of Malaysia, Kuala Lumpur 50300, Malaysia; 3 School of Public Health, College of Health, Adelaide University, Adelaide, SA 5005, Australia; lyle.palmer@adelaide.edu.au; 4 Australian Institute for Machine Learning, Adelaide University, Adelaide, SA 5005, Australia

**Keywords:** anthropology, odontology, geometric morphometrics, palate, population affinity, semi-landmarks

## Abstract

**Background/Objectives**: This study evaluated 3D palatal shape variation to distinguish population affinities, aiming to enhance forensic and anthropological assessments of unknown human remains. **Methods**: Maxillary dental casts (*n* = 100 per group) from four population groups, Australian Europeans, Malaysian Malays, Malaysian Chinese and Malaysian Indians, were digitised using a 3D scanner. Fourteen gingival and two mid-palatal landmarks and 99 surface points (semi-landmarks) were placed to capture the overall palatal shape. Shape data were aligned using Generalised Procrustes Analysis (GPA) followed by Principal Component Analysis (PCA) to explore shape variation. Canonical Variate Analysis (CVA) and Mahalanobis distances were used to assess group differences. Classification performance was evaluated using accuracy, sensitivity, specificity, precision, and F1 score. **Results**: The first five principal components (each explaining ≥5% of the observed variation) were retained for CVA. CV1 accounted for 88% of the between-group variance, showing clear separation of Europeans from the clustered Malay–Chinese group, with Indians appearing intermediate. European palates were longer, narrower, deeper, and more anteriorly tapered. Significant shape differences were found between all groups, except between Malays and Chinese. The overall classification accuracy was 45%, with better performance for European and Indian groups. Specificity was higher across all groups (0.70–0.90), while sensitivity, precision, and F1 scores were moderate. **Conclusions**: Geometric morphometric analysis revealed population-level differences in palatal shape, with moderate accuracy and high specificity. These findings support its role as a complementary tool alongside other data in forensic and anthropological applications. While palatal shape alone cannot definitively classify populations, it may be useful for excluding certain groups.

## 1. Introduction

The morphology of the human palate shows significant variation across different populations, influenced by a complex interplay of genetic, environmental, and developmental factors [1,2]. Understanding this variation is important for both clinical and forensic applications. Clinically, palatal shape influences orthodontic diagnosis and treatment planning, prosthodontic outcomes and airway-related conditions, supporting more individualised dental care. Population-specific palatal morphology may also provide insight into the aetiology of conditions such as orofacial clefts, which shows variation in prevalence across populations. From a forensic perspective, population-specific variation in palatal morphology can contribute to biological profiling and the identification of unknown remains.

Estimating human ancestry is a key aspect for developing a biological profile, which involves studying the morphological characteristics of anatomical structures that are assumed or known to be derived from shared genetic or environmental factors and categorising them into specific groups. For repatriation of unidentified human remains, biological profiling is crucial. Ancestry refers to the biological origins of an individual, which extend beyond self-reported race or ethnicity [3]. While race and ethnicity represent social or cultural constructs that can vary by country and may change over time based on personal self-identification, ancestry traces an individual’s biological roots through genetic, genealogical, and geographical factors [4]. It is, however, important to recognise that ancestry classification should be viewed as a continuum reflecting clines of variation without clear-cut boundaries [5]. Broad geographical or continental groupings, such as Africans, Asians, and Europeans, are commonly used in research studies, as these groups share common ancestors and histories spanning many generations [6,7,8]. Additionally, further subdivisions into sub-continental groups, such as Central/South Asia and East Asia, are commonly employed, provided they align with genetic and historical clustering [6,7,8]. While the concept of ancestry may hold less relevance for understanding the complex genetic diversity of contemporary populations, it remains a valuable tool in the repatriation and identification of historic human remains, where genetic and morphological patterns often reflect more distinct ancestral lineages. Acknowledging the fluidity of human variation and the limitations of rigid categorisation, the concept of population affinity is often employed in forensic anthropology as it allows for a more flexible and context-sensitive interpretation of biological relationships [9]. Accordingly, population affinity will be the preferred terminology used in this paper, rather than ancestry or race.

While DNA-based methods have advanced estimation of population affinity, they are not always applicable, particularly when remains are highly degraded [10]. Additionally, obtaining DNA samples is more destructive and may not be ideal for fragile and rare specimens [10]. Furthermore, the invasive nature of DNA sampling may not be culturally acceptable for certain groups, such as Indigenous communities. In such cases, anatomical methods remain essential. These approaches are not only less destructive but also more cost-effective and practical, especially when dealing with large sample sizes. Conducting DNA analysis on every specimen would require significant time, resources, and specialised facilities, making morphological assessment of anatomical structures a more feasible method in many contexts.

Morphoscopic analysis is the method commonly used by anthropologists to visually assess non-metric traits, categorise them into presence/absence or degree of expression, and then apply them to estimate population affinity [11]. The skull is the most useful skeletal structure to indicate population affinity, with up to 92% classification accuracy, based on the morphology of structures like the nasal cavity and orbit [12,13]. Within the oral cavity, the maxillary arch shape has been described as parabolic, hyperbolic and elliptical in European, African, and Asian populations, respectively [14,15]. Although morphoscopic methods have been widely used in forensic anthropology for population estimation, their limitations, particularly regarding subjectivity, lack of standardisation, and poor reproducibility, highlight the need for more robust alternatives [16,17]. Traditional morphoscopic analyses rely on the frequency of non-metric craniofacial traits and use decision-tree or scoring models to make probabilistic population classification, which can oversimplify complex biological variation underlying population differences and often lack clear developmental or functional explanations. These binary or categorical classifications are prone to observer bias and can vary depending on the examiner’s training and experience [18].

To overcome the limitations of traditional morphoscopic analysis, geometric morphometrics provides a quantitative approach for capturing continuous shape variation using two-dimensional or three-dimensional landmark-based data [19]. Unlike conventional linear measurements, this approach preserves the spatial configuration of landmarks, enabling the application of multivariate statistical techniques for the robust quantification, visualisation, and hypothesis testing of shape differences [19,20]. Geometric morphometrics allows for more detailed, objective, and reproducible assessments of craniofacial morphology [21,22,23]. Furthermore, it facilitates the analysis of the entire geometry of anatomical structures, including subtle shape variation that may not be detectable or classifiable using discrete trait categories [23]. A key advantage of this method is its capacity to distinguish between shape and size, allowing for independent analyses of each component, and the investigation of allometric relationships [24].

Geometric morphometric approaches have been applied in past studies to study palatal shape variation across different populations. For instance, Badawi-Fayad et al. [25] utilised geometric morphometrics analysis of craniofacial variation, finding that the length and relative inclination of the palate were some of the useful features for distinguishing between European, American and African populations. However, this study was based on skeletal collections dated to the 19th century, which may not accurately represent contemporary populations, particularly given increasing levels of admixture. Similarly, Clark et al. [26] also applied geometric morphometrics to study variation in the maxillary dental arch, but a lack of good discrimination between the Gullah (descendants of West Africans) and the Seminole (Indigenous Americans) was observed, with a reported accuracy of just 62% using discriminant function analysis (DFA). The contrast between both studies could be due to the relative genetic distance of the populations studied in the two different studies. In contrast, El Segani et al. [27] found significant shape differences in pairwise group comparisons of European, Asian and African populations using Canonical Variate Analysis (CVA), although the predictive accuracy of the model was not reported. A limitation in the existing literature relates to sampling strategies. Many studies have unbalanced sample compositions, with an over-representation of European samples and relatively fewer African and Asian individuals [28]. Furthermore, sample classification is often based on traditional concepts which may not align with current understandings of population affinity. These factors introduce uncertainty regarding the “ground truth” of group assignment and may contribute to inconsistencies in reported findings.

Another limitation in many earlier studies is insufficient landmark coverage on the palatal surface [25,26,27], as well as the reliance on two-dimensional (2D) analyses [26]. These constraints often fail to capture the complex three-dimensional (3D) morphology of the palate. As a result, it remains unclear whether the inconsistent findings in previous studies reflect a lack of genuine biological differences between populations or methodological limitations in capturing palatal morphology [28]. In particular, these inconsistencies may arise from inadequate three-dimensional representation of the palatal surface due to the limited number of identifiable fixed landmarks. Given these challenges, it is worth exploring whether palatal shape features may have previously been obscured by the lack of landmarks, and whether the predictive accuracy of using palatal shape to estimate population affinity could be improved by employing 3D analysis with a denser set of landmarks. Advances in digital scanning technology, including full facial surface scanners and intraoral/extraoral dental scanners, have enabled reproducible 3D analysis of craniofacial and palatal structures, demonstrating their potential utility in both dental and forensic applications [29,30,31,32]. However, the accuracy of linear measurements may vary depending on the specific technology employed, with some studies reporting reduced accuracy for larger measurements (e.g., >65 mm) [32].

The current study, therefore, aimed to evaluate 3D palatal shape variation among several different populations for which our group has available data: Australian Europeans, Malaysian Malays, Malaysian Chinese, and Malaysian Indians. The objectives were to assess the discriminative and predictive potential of three-dimensional palatal shape in distinguishing between these populations and to identify specific shape features that may aid in differentiating population affinities.

## 2. Materials and Methods

### 2.1. Sample Population

We undertook a retrospective cross-sectional study of two different collections of anonymised plaster dental casts housed in the Adelaide Dental School at the University of Adelaide, Adelaide, Australia. Written informed consent was obtained from all participants for the collection of dental casts, as approved by the University of Adelaide Human Research Ethics Committee in previous studies (HREC 07-1984A, HREC 27-1990, HREC 78-2003 and HREC 2013-097). For this study, ethics approval was obtained from the University of Adelaide Human Research Ethics Committee to use the collections of dental casts (H-2024-134, approval date: 23 September 2024).

The first dataset was derived from participants in “Dentofacial variation in twins: genetic and environmental determinants”, a longitudinal study undertaken of twins of European descents from 1995 to 2006, with dental casts collected in the primary, mixed and permanent dentition stages [33]. The casts for permanent dentition were fabricated when the participants were 14.5 ± 1.1 years. Only permanent dental casts of one of the members of each twin pair (randomly selected) were used to represent European population. The second dataset was derived from a previous cross-sectional study by Khamis and colleagues, which examined dental variation in the Malaysian population [34]. Dental casts of the permanent dentition, collected in 2003, were available from participants of Malay, Chinese and Indian populations in Malaysia, when they were 15.2 ± 1.3, 14.5 ± 1.4 and 15.4 ± 1.4 years, respectively.

Although a convenience sample for the purposes of this study, the selected sample populations reflect, to some extent, the diversity of Australia’s broader demographic profile. According to the 2021 Australian census, individuals of North-West European descent comprise approximately 46% of the population, followed by those of Southern and Eastern European (11%), South-East Asian (4%), North-East Asian (6%), and Southern and Central Asian (7%) backgrounds [35]. While the current study focuses on individuals of European and selected Asian populations (Malay, Chinese, and Indian), these groups together represent a substantial proportion of Australia’s diverse population. As such, the inclusion of these populations enhances the relevance of the findings to the Australian context and contributes to the understanding of dentofacial variation within and between population affinities.

### 2.2. Selection Criteria

The inclusion criteria were:

- Healthy individuals (i.e., all the study participants were free from any craniofacial syndromes or anomalies, including cleft palate, and had no history of maxillofacial trauma and no prior surgical treatment that could affect palatal morphology);
- Permanent dentition;
- Presence of all teeth from Fédération Dentaire Internationale (FDI) tooth numbers 17 to 27 [36].

Dental cases were excluded based on the following criteria:

- Poor quality casts or casts that contained significant artefacts on landmark locations and/or the palatal surface;
- A history of past orthodontic treatment;
- Dental anomalies, such as hypodontia and supernumerary teeth.

Maxillary casts that fulfilled the selection criteria (*n* = 100 per group) were randomly selected for each population affinity—Australian Europeans, Malaysian Malay, Malaysian Chinese and Malaysian Indian. A total of 400 dental casts (*n* = 400) were thus included in the current study. There was an equal number of males and females in each population sample. Although a formal power analysis was not performed, sample size adequacy was considered in relation to effect sizes reported in a previous study [27]. The present sample size is consistent with ranges typically required for stable discrimination of moderate multivariate shape differences and was additionally constrained by the availability of specimens meeting the inclusion criteria.

The dataset was randomly divided into training and testing subsets, stratified by population group. Ninety percent of the samples (*n* = 360) were used for model training and cross-validation, while the remaining ten percent (*n* = 40) were reserved as an independent testing dataset to evaluate the classification performance of the model.

### 2.3. Digitisation of Dental Casts

All dental casts were scanned using an extra-oral 3D scanner (3Shape, E4, Copenhagen, Denmark) with an accuracy of 4 µm. The digital casts were then stored in standard tessellation language (.stl) format.

### 2.4. Landmarking

The digital scans were imported into an open-source biomedical visualisation software, 3D Slicer (version 5.6.2; Surgical Planning Laboratory, Brigham and Women’s Hospital, Boston, MA, USA), for landmark acquisition using the Markups Tool [37,38,39].

Fourteen periphery (gingival) landmarks and 2 midline (mid-palatal) fixed landmarks were used. Landmarks 1 to 14 represent the deepest points of the palatal gingiva corresponding to each maxillary tooth, from the right second molar (FDI 17) to the left second molar (FDI 27). Using the 14 gingival landmarks, a gingival plane was defined through a singular value decomposition of the palatal gingiva of all 14 maxillary teeth. Similarly, an orthogonal mid-palate plane was derived, and then, transverse planes orthogonal to both the gingival and mid-palate planes were created. These were based on the relevant gingival antimeres projected onto the gingival plane. The two mid-palatal landmarks (landmark 15: first premolar region; landmark 16: first molar region) were then established at the intersection of the mid-palatal plane, the relevant transverse plane (first premolars or first molars), and the surface mesh of the .stl file.

**Figure 1:**
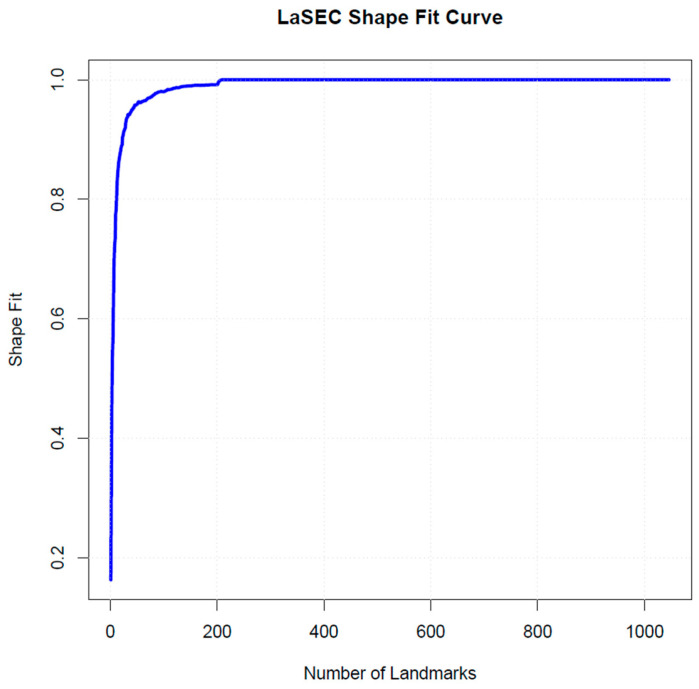
Landmark Sampling Evaluation Curve (LaSEC) showing the relationship between the number of landmarks and shape fit. The curve demonstrates a rapid increase in shape fit with the addition of landmarks, followed by a plateau at 100–120 landmarks, indicating diminishing returns in capturing additional morphological detail.

**Figure 2:**
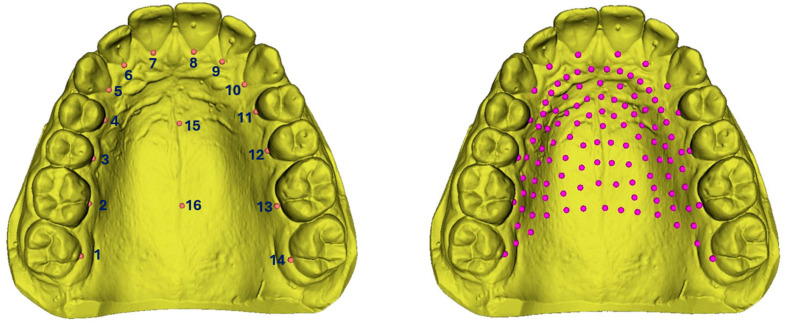
Palatal landmarks and semi-landmarks. Fourteen gingival landmarks on 17 to 27 (labels 1 to 14); two mathematically computed mid-palatal landmarks (labels 15 and 16); semi-landmarks on the palatal surface (purple).

Semi-landmarks were then placed between the user-specified anatomical landmarks using the CreateSemiLMPatches module of SlicerMorph extension in 3D Slicer to capture the shape of palatal surfaces [40]. Semi-landmarks refer to the points used in geometric morphometrics to capture the shape of curves and surfaces where true anatomical landmarks are not clearly defined [19,41]. While not biologically homologous in the traditional sense, semi-landmarks are placed between or along anatomical boundaries defined by fixed landmarks and are constrained to slide along those curves or surfaces [42]. This sliding process optimises alignment across specimens and ensures that the variation captured reflects true morphological differences rather than arbitrary point placement [42]. To determine an appropriate number of semi-landmarks required to adequately capture palatal morphology, a Landmark Sampling Evaluation Curve (LaSEC) was generated [43]. This curve assesses the relationship between the number of landmarks and the corresponding shape fit, allowing identification of the point at which additional landmarks provide diminishing returns in capturing morphological variation. As shown in Figure 1, the shape fit increased rapidly with the initial addition of landmarks and approached a plateau at approximately 100–120 landmarks. Beyond this point, further increases in landmark number resulted in minimal improvement in shape representation. Based on this plateau, a total of 115 3D landmarks (16 fixed landmarks and 99 evenly allocated semi-landmarks) were retained for subsequent geometric morphometric analyses, ensuring an optimal balance between shape fidelity and computational efficiency. Figure 2 illustrates the landmarking procedure. Consequently, all samples contained similar landmark configurations, including homologous anatomical landmarks and standardised semi-landmarks, suitable for geometric morphometric analysis.

### 2.5. Statistical Analysis/Geometric Morphometrics

For statistical analysis, the 3D landmark data were imported into the R software environment (version 4.4.1; R Core Team, Vienna, Austria), implemented within RStudio (version 2024.04.2; Posit Software, PBC, Boston, MA, USA).

A Generalised Procrustes Analysis (GPA) was performed on the landmark data using the procSym() function from the Morpho package (version 2.13) in R [44,45]. This method standardises landmark configurations by removing variation due to translation, rotation, and scale. As a result, all data were aligned to a common coordinate system, known as Procrustes coordinates, that represent a shape dataset independently of isometric size, suitable for further multivariate analyses. Following GPA, the relationship between shape and size (allometry) was assessed by regressing Procrustes-aligned coordinates against centroid size. When significant allometric effects were detected, shape data were corrected for allometry by using the residuals of this regression.

Principal Component Analysis (PCA) was performed on the aligned landmark coordinate data to reduce dimensionality and identify major axes of shape variation [19,46]. PC scores were extracted directly from the PCscores component of the procSym output. The PCA summarises variation in the shape data by orthogonal axes, known as principal components (PCs). Each PC represents a specific pattern of variation in shape. The first PC captures the largest amount of variation present in the dataset, while each subsequent PC explains a progressively smaller amount of the remaining variation. PCs that explained at least 5% of variance were retained for subsequent discriminant analysis and group comparisons, complemented by visual inspection of the shape deformations associated with each principal component to ensure interpretability [47].

Canonical Variates Analysis (CVA) was then performed to assess shape differences among the four population groups (defined a priori) based on the retained principal components [48,49]. The retained PCs derived from prior PCA were used as input to CVA, which assigns different weights (loadings) to each PC to maximise the ratio of between-group to within-group variance. This process generates canonical axes (CVs) that optimally discriminate among the groups. Visualisation of shape changes along these axes was performed to facilitate biological interpretation of the group differences. Pairwise Mahalanobis distances were also calculated between group centroids in the canonical variate space to quantify shape differences [49,50]. To assess statistical significance, permutation tests with 1000 iterations were performed, where group labels were randomly reassigned to generate an empirical null distribution of Mahalanobis distances. Observed values were compared to this distribution, and *p*-values were computed for each pairwise comparison. Statistical significance was set at *p* < 0.05. Classification accuracy was assessed using leave-one-out cross-validation and further evaluated using an independent testing dataset to examine the robustness of group discrimination. The classification performance was evaluated using the metrics defined in Table 1.

### 2.6. Method Error

A single examiner identified the landmarks on all digital dental casts following a standardised landmarking protocol with clearly defined anatomical criteria. Prior to data collection, two examiners underwent calibration training, which involved joint review of landmark definitions and practice landmarking sessions to ensure consistency in landmark placement. Landmark identification was repeated by the primary examiner after a four-week interval on 20 randomly selected casts to assess intra-examiner reliability. A second examiner independently landmarked the same 20 casts to assess inter-examiner reliability. The degree of method error was estimated using Procrustes ANOVA, which is appropriate for multivariate, spatially correlated shape data in geometric morphometrics [51]. Unlike intra-class correlation (ICC), which assumes univariate and independent data, Procrustes ANOVA operates directly on Procrustes-aligned landmark configurations and partitions variance in shape space.

## 3. Results

### 3.1. Reliability Analysis

The method error for intra-examiner variation accounted for 0.2% of the total variation, suggesting that the repeatability of the landmarking process was excellent. The inter-examiner error accounted for 1.5% of the total variation, which also indicated a low level of variation between examiners. Both intra- and inter-examiner variation were low, demonstrating a good level of consistency and reliability in the landmarking process.

### 3.2. Effects of Population on Palatal Shape

Procrustes ANOVA (Table 2) revealed a significant effect of population on palatal shape, while sex also had a small but statistically significant effect. The absence of a significant population:sex interaction suggests that the influence of sex on palatal shape is consistent across populations, allowing population differences to be interpreted without evidence of sex-dependent effects/bias.

### 3.3. Allometric Effects on Palatal Shape

Procrustes ANOVA revealed a significant relationship between palatal shape and centroid size (R^2^ = 0.04, F = 15.62, *p* < 0.01), indicating the presence of allometry. However, centroid size accounted for a relatively small proportion of total shape variation (4.2%).

**Figure 3:**
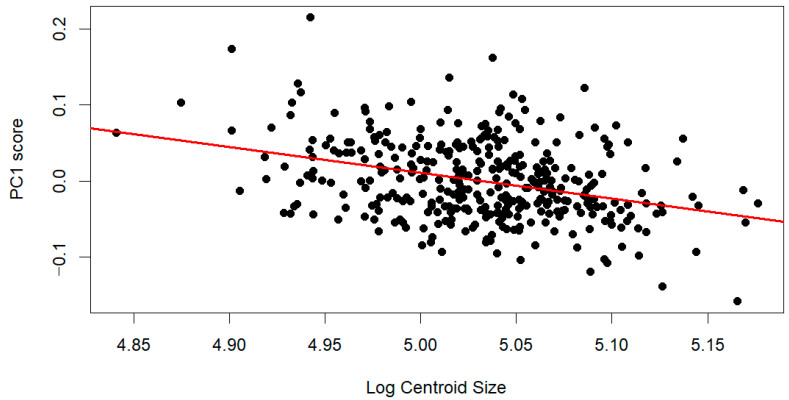
Scatterplot of log-transformed centroid size against PC1 scores. Points (black) represent individual specimens, and the fitted regression line (red) indicates a weak negative relationship, suggesting that size-related shape variation is partially aligned with the primary axis of palatal shape variation.

A scatterplot of log-transformed centroid size against PC1 scores showed a weak negative association, suggesting that larger individuals tend to exhibit lower PC1 scores (Figure 3). This indicates that size-related shape variation is partially aligned with the primary axis of shape variation, although the relationship is modest.

Given the significant allometric effect, subsequent analyses were performed using shape variables corrected for allometry.

### 3.4. Principal Component Analysis (PCA)

**Figure 4:**
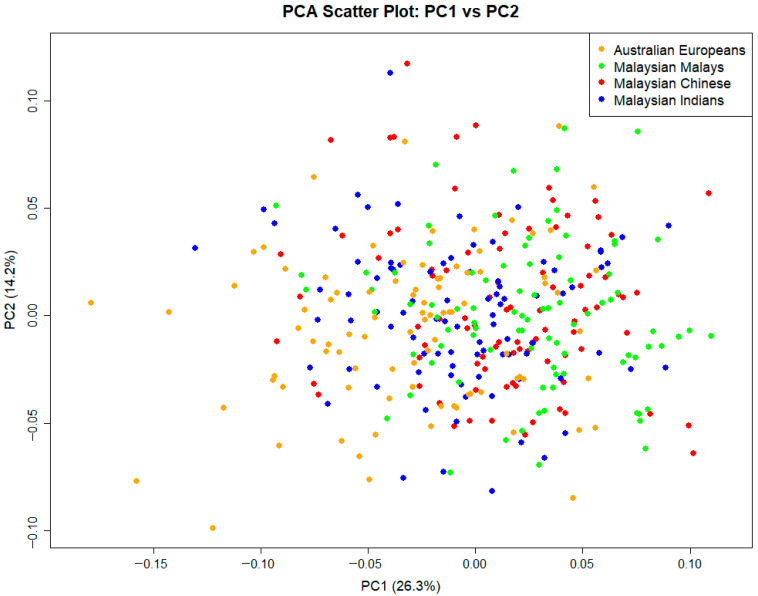
PC1 vs. PC2 scatterplot showing palatal shape variation among the four population groups.

Principal Component Analysis (PCA) explored the total shape variation in the entire sample. The first five PCs each explained at least 5% of the total shape variation and cumulatively explained 61% of the total shape variation. Table 3 depicts the eigenvalues and percentage variance explained by each PC. In the PCA plot of palatal shape variation (Figure 4), a high degree of overlap was observed among the four population groups, reflecting the continuous nature of human morphological variation. However, some general trends were evident along the first principal component (PC1). European individuals tended to cluster toward the negative end of PC1, while Chinese and Malays were more commonly positioned toward the positive end. Indian individuals were distributed more centrally, overlapping with all other groups.

**Figure 5:**
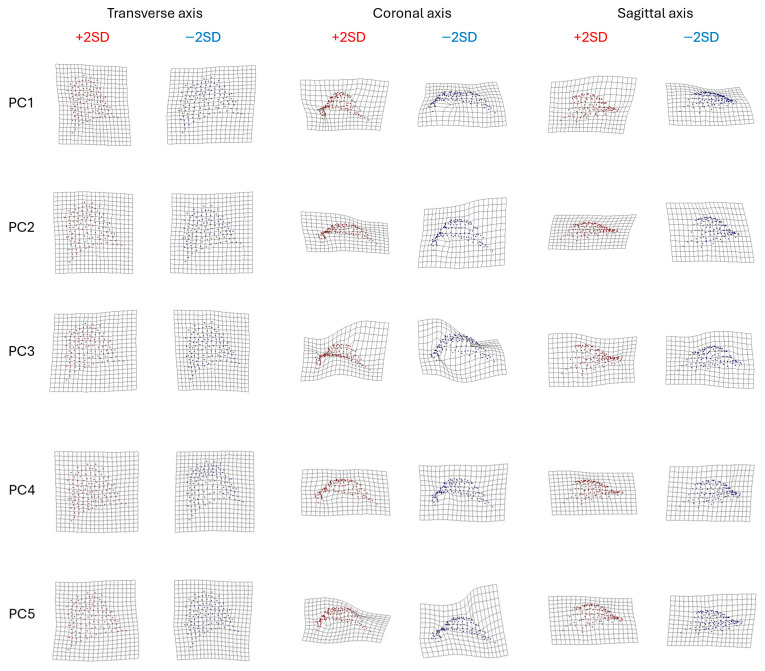
TPS 2D deformation grids for the first five PCs, with blue representing −2SD and red representing +2SD.

Visualisation of shape variation for each PC using Thin-Plate Spline (TPS) 2D deformation grids, in the XY (transverse) plane, XZ (coronal) plane and YZ (sagittal) plane, at ±2 standard deviations (SD), revealed complex variation in shape of the palate at different sites (Figure 5). Descriptions of the first five PCs are provided in Table 4.

### 3.5. Canonical Variate Analysis (CVA)

The first five PCs were retained for subsequent multivariate analysis by Canonical Variate Analysis (CVA), which aims to maximise between-group shape variation relative to within-group variation. Unlike PCA, which is unsupervised and can generate as many components as there are variables, CVA is a supervised method that uses a priori group information and can produce at most one fewer axis than the number of groups. In this study, CVA generated three canonical variates (CVs) for four groups, with each axis representing a direction that maximally separates the groups. Table 5 summarises the variance explained by each CV.

Table 6 presents the canonical variate (CV) loadings for the first five PCs, showing their relative contributions to CV1, CV2, and CV3. Higher absolute values indicate stronger influence of a given PC on the corresponding CV axis.

**Figure 6:**
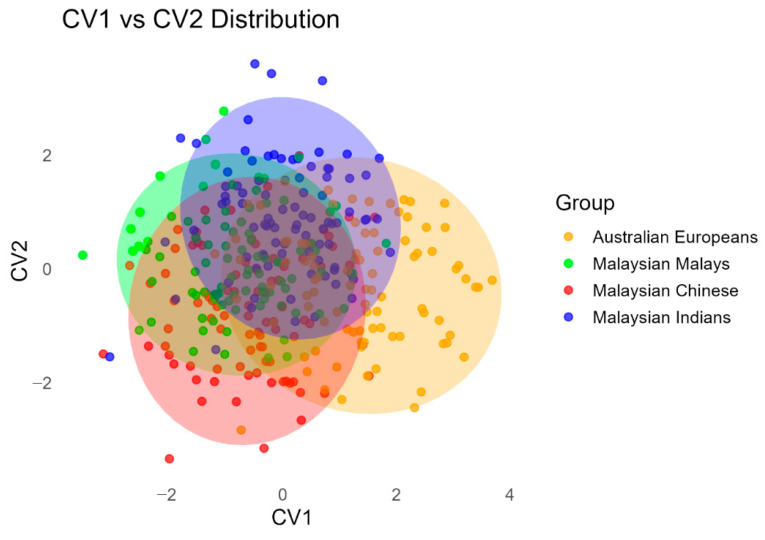
CVA scatterplot between CV1 and CV2, showing group separation by population. Each point represents a specimen, coloured by group. The shaded ellipses represent 95% confidence intervals for each group, indicating the region where 95% of the group’s specimens are statistically expected to fall under a multivariate normal distribution.

**Figure 7:**
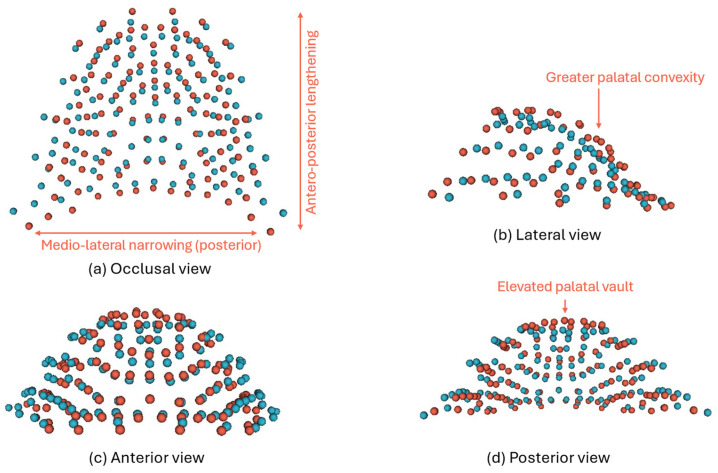
Visualisation of 3D changes in CV1 (**a**) occlusal view; (**b**) lateral view; (**c**) anterior view; and (**d**) posterior view. Blue represents −2SD and red represents +2SD. Descriptions are labelled to describe the variation at +2SD.

The results from CVA indicated that most of the shape differentiation between groups was captured along the first canonical variate (CV1), which accounted for 88% of the total between-group shape variance, and represented the primary axis of discrimination. Visual inspection of CVA scatterplots showed some degree of overlap among the groups, but emerging trends in shape separation were evident (Figure 6). Higher CV1 scores are associated with increased palatal concavity, characterised by a deeper and more arched palatal vault, a relatively narrower arch posterior to the canines and an antero-posterior elongation of the arch (Figure 7). The vault is more elevated, giving the impression of an extruded or more vertically developed palatal contour. The shape of the palatal arch is more tapered (V-shaped) due to the anterior elongation and posterior narrowing. Conversely, lower CV1 scores reflect a flatter (less concave) palate with a localised depression at the central palatal vault, a broader posterior arch, a more rounded arch shape, and a relative antero-posterior shortening. CV1 may be interpreted as reflecting a pattern of shape variation broadly associated with differences in population affinity among the sampled groups:

- The Malay and Chinese samples, representing population affinity groups within a broader Asian context, clustered together on the negative side of CV1, indicating similar palatal shape characteristics.
- The European sample was positioned on the positive side of CV1.
- The Indian sample occupied an intermediate position along CV1, suggesting overlapping morphological features with both the European and Malay-Chinese groups.

**Figure 8:**
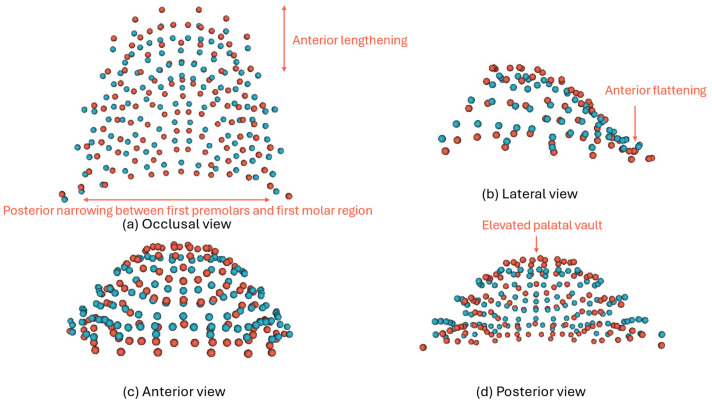
Visualisation of 3D changes in CV2 (**a**) occlusal view; (**b**) lateral view; (**c**) anterior view; and (**d**) posterior view. Blue represents −2SD and red represents +2SD. Descriptions are labelled to describe the variation at +2SD.

Along CV2, which accounted for 9% of variation, higher scores were associated with lengthening and flattening of the anterior palate combined with a deeper palatal vault. The palate had constriction/narrowing between the first premolar and first molar region and widened at the second molar region, giving an hour-glass appearance to the palatal arch (Figure 8). CV2 contributed to the partial separation between Indian and Malay samples.

Pairwise Mahalanobis distances revealed significant shape difference between Australian European and all three Malaysian populations (Table 7). The Malaysian Indian group showed relatively smaller differences from the other three groups, yet it was still significantly separated in shape space. The Mahalanobis distance between the Malaysian Chinese and Malaysian Malay indicate that the difference between both populations was insignificant. Despite statistically significant Mahalanobis distances between most groups, the considerable overlap of group distributions indicates that palatal shape variation among the populations is moderate and not clearly distinct at the individual population level.

Classification using CVA yielded an overall classification accuracy of 49%, indicating moderate agreement beyond chance. However, accuracy decreased to 44% following cross-validation and 45% in the independent test dataset, suggesting some degree of overfitting and reduced generalisability of the model. Confusion matrices for the original, cross-validated, and test classifications, as well as overall model performance, are presented in Table 8.

The classification results in the test dataset indicate varying levels of performance across the four population groups. Sensitivity (recall) values ranged from 30% for the Malay population to 60% for the European population, suggesting that the model was most effective at correctly identifying European individuals and least effective for Malay individuals. Specificity was generally high across all groups (70% to 90%), indicating the model’s strong ability to correctly exclude non-members of each group. Precision values ranged from 25% (Malay) to 67% (European), reflecting the proportion of correctly predicted cases within each predicted group. The F1 scores, which balance precision and recall, further highlight the moderate classification performance, with the European and Indian groups performing better (0.63 and 0.53, respectively) compared to Malay (0.27) and Chinese (0.40).

## 4. Discussion

This study used three-dimensional geometric morphometrics with semi-landmarks to explore population-level variation in palatal shape across four population affinities. By capturing detailed surface geometry of the palate, followed by Generalised Procrustes Analysis (GPA), this approach enabled a focused analysis of shape variation, independent of size, orientation and position.

### 4.1. Findings from PCA

In Principal Component Analysis (PCA), the first principal component (PC1) typically represents the greatest axis of variation within the dataset. In this study, PC1 only explained 26% of the variation, which is not particularly high. This is because this study focused specifically on shape differences after subjecting all landmark configurations to Generalised Procrustes Analysis (GPA) and allometry correction, which remove non-shape variation. Consequently, the proportion of variance explained by PC1 in shape space is generally lower than in analyses that include size, where PC1 may dominate due to allometric effects. Furthermore, a dense semi-landmark configuration was designed to capture the complex 3D morphology of the palatal surface, which lacks sufficient fixed anatomical landmarks for precise representation. Semi-landmarks enable detailed quantification of subtle curvature, asymmetry, and surface variation relevant to inter-population shape differences [52]. In contrast to fixed landmarks, which are limited to discrete anatomical points, semi-landmarks allow a more comprehensive and biologically meaningful assessment of shape [41]. Semi-landmarks increase dimensionality and capture more orthogonal shape modularity, distributing variance across more PCs. However, this added complexity is necessary to enhance the resolution for detecting subtle morphological differences.

The pattern of shape variation observed in PCA suggests that variation in one region of the palate often covaried or was coupled with changes in other regions, suggesting interdependent growth relationships. For instance, along PC1, a higher palatal vault was associated with a longer and narrower palatal arch. This type of integrated shape variation aligns with findings by El Sergani et al. [27], who reported that a higher palatal vault was associated with a shorter palate in their first PC. Likewise, PCA in patients with Class II skeletal pattern also revealed a high palate associated with a long and narrow palate [53]. Another study on Class III skeletal pattern subjects also had associated high palate and narrow palate from PCA [54]. This internal coordination supports the idea that different regions of the palate develop in a morphologically and functionally interdependent manner, likely reflecting shared developmental mechanisms or mechanical constraints during craniofacial growth.

The PCA results revealed substantial overlap among the four population groups, particularly between Malaysian Chinese, Malays, and Indians, which is consistent with the expected clinal nature of human morphological variation. However, subtle trends were observed, with European individuals tending to cluster toward the negative end of the first principal component, while Chinese and Malay individuals appeared more on the positive side, and Indians were distributed centrally. Despite the lack of clear group separation, these emerging patterns suggest some degree of population-level structure in palatal morphology. However, this variation was not sufficient for strong group differentiation using PCA alone.

### 4.2. Findings from CVA

The first CV appears to capture patterns of palatal shape variation associated with differences in population affinity, with the European sample differentiated from the three other Malaysian groups. Given their close geographic proximity, the Malay, Chinese, and Indian populations sampled from Malaysia may be considered distinct population affinity groups within a broader Asian regional framework, alongside a European population sampled for comparison. Hence, the results align with findings from El Sergani et al., which also reported that Europeans generally exhibit longer and narrower palates compared to Asians [27]. Our study also supports the observation that Asian populations tend to have shallower palatal vaults relative to Europeans, consistent with findings by El Sergani et al. [27].

The palatal arch shape demonstrated in this study fits the earlier description of the arch shape of European and Asian as parabolic and elliptical, respectively [14]. Past studies of maxillary arch curvature using fourth-order orthogonal polynomial analysis also showed that the European population has a more tapered arch, suggesting a tendency for a more parabolic-shaped arch [55]. Furthermore, the localised dip or intrusion of the superior contour of the mid-palate, noted at the Malay-Chinese end of CV1, can be one of the palatal traits useful to discriminate populations. This is supported by the fact that torus palatinus (a bony protrusion often seen on the midline of the hard palate) is more commonly observed among Asian than European populations and is associated with a wider and shorter palate [56].

CVA showed that the Indian group occupies an intermediate position between the Chinese and Malay groups on one side and the European population on the other. These findings align with the understanding of how Asia was populated. The Indian group (Central/South Asian population) reflects a genetic and morphological position intermediate between populations with stronger East Asian population (Malay and Chinese) and those with European population, consistent with South Asia’s role as a human migratory corridor and a genetic bridge between East Asia and Europe [57]. Pairwise comparisons of group centroids using Mahalanobis distances also showed that the Indian population was distinct from all three other populations. While all three Malaysian groups were broadly classified as Asians, the Indian population in Malaysia may exhibit craniofacial traits that partially overlap with those observed in the European populations. A study on dental arch shape using 2D analysis also found that Indian and European populations exhibited a closer relationship to each other, while Malay and Chinese populations were more closely related [55]. Using craniofacial measurements derived from post-mortem computed tomography (PMCT) images, a recent study reported higher classification accuracy for Indian crania compared to their Malay and Chinese counterparts [58]. This is likely due to the presence of distinctive craniofacial traits that more reliably differentiate Indian individuals from the other two population affinity groups [58]. This finding may be attributed to the similarity of certain craniofacial traits between Indian and European populations (such as narrow interorbital width, narrow nasal width and mesocephalic skull), which could explain why the palatal shape of the Indian group appeared intermediate between the Malaysian Malay and Chinese groups and the Australian European group in the present study [58].

Interestingly, asymmetrical features of the palate were observed using PCA but were less evident in the CVA, suggesting that it reflects individual-level variation rather than a consistent population-level pattern. PCA captures total shape variance, including within-group variation and non-directional asymmetries, making it sensitive to subtle individual deviations. In contrast, CVA maximises between-group differences while minimising within-group variance, which may reduce the visibility of individual-level asymmetry. The absence of strong asymmetry effects in the CVA therefore supports the interpretation that these shape differences are not population-specific but instead reflects individual morphological variation, which may still be useful in forensic contexts for individual identification when assessing remains or comparing postmortem records to antemortem data [59].

### 4.3. Predictive Performance of the Model

While the overall classification accuracy in this study was 45%, this is notably higher than the expected chance level of 25% for a four-class model. However, it should be noted that the independent test set was relatively small (*n* = 40), which may limit the stability and precision of the performance estimates. These findings therefore reflect a preliminary assessment of model performance and should be interpreted with some caution and also reflect the complexity of palatal shape variation across the populations analysed.

These findings are consistent with previous research that has reported classification performance based on palatal shape, although, notably, most of these studies have relied solely on palatal arch curvature rather than the entire palatal surface. For example, Clark et al. [26] reported a modest classification accuracy of 68% in a two-group comparison between African American and Indigenous American populations. Similarly, Maier et al. [14] achieved an accuracy of 58% when classifying American individuals into four groups (White, Black, Asian/Native American, and Hispanic). To date, there appear to be no published studies that evaluate classification performance using the full palatal shape, despite several having explored shape variation among different populations descriptively.

The classification results indicate that the model demonstrates relatively high specificity across all groups (ranging from 0.70 to 0.90), suggesting it was effective at correctly identifying when a sample did not belong to a particular group. However, the lower sensitivity (recall) values, particularly for the Malay (0.30) and Chinese (0.40) groups, indicated that a substantial number of true positives were missed. The low precision for these groups also highlights the model’s limited ability to correctly assign group membership, with a considerable proportion of false positives. These patterns suggest that while the model could effectively rule out group membership, it struggled with correctly classifying samples, especially when shape differences were subtle or overlapping between groups. Given the high specificity demonstrated by the model, however, there is potential utility in anthropological and forensic contexts where the objective is to exclude certain group memberships. For instance, the model could support expert judgement by indicating that a sample is unlikely to belong to a particular population. This kind of exclusion-based inference can be particularly useful in narrowing down the potential populations of unknown remains during identification processes. While the model may serve as a supportive tool for exclusion, it should not be used in isolation for definitive estimation of population affinity. Instead, caution is required when interpreting positive classifications, and we recommend integrating with other lines of evidence (e.g., additional morphologies, genetic data, contextual information) for a robust conclusion.

### 4.4. Limitations and Recommendations

The current study highlighted the strength of using dense three-dimensional semi-landmarks to capture and visualise subtle variations in palatal morphology across different population groups. This approach enables detailed shape analysis beyond the limitations of traditional linear metrics. However, several limitations of our study should be acknowledged. First, our study focused on only four different population affinities, limiting broader generalisability; inclusion of individuals of Indigenous Australian and African populations would provide a more comprehensive picture of palatal variation and provide a comparative reference for these populations. Secondly, although a 90/10 training–testing split was used, the small size of the test dataset (*n* = 40) limits the robustness of the validation and may reduce confidence in the reported performance. Estimates derived from small test samples are more prone to variability and may not fully reflect model generalisability or predictive analyses. A larger sample size, and consequently a larger test set, would improve the stability of performance metrices and reduce the risk of overfitting. Thirdly, potential environmental or behavioural influences on palatal shape, such as thumb-sucking, pacifier use, or mouth-breathing, were not recorded, although they are known to influence palatal development and may either inflate or reduce estimated population affinities.

Although semi-landmarks allow for high-resolution shape capture, the shape differences observed in the CVA were not particularly striking, suggesting that the groups under comparison may share a high degree of morphological similarity or that the variation is subtle and not easily detected visually. This highlights a broader challenge in anthropology: the limitations of relying solely on visual assessment for classification and interpretation. Subtle shape variations, especially in complex 3D structures, often go unnoticed without computational support. The digitisation of specimens and the application of geometric morphometric methods in this study enable more precise and quantitative examinations of palatal shape. Even when visual differences appear minimal, statistical techniques in this study revealed patterns of variation that would otherwise be overlooked. The moderate classification accuracy highlights the difficulty of using palatal morphology alone for reliable group differentiation and suggests that incorporating additional features (e.g., palatal size, or neighbouring structures such as teeth) or more advanced modelling techniques (e.g., deep learning) may be necessary. While palatal morphology may provide useful information for estimating population affinity, applying this approach in practice could be made more user-friendly and streamlined. This would require the development of an appropriate database or software programme, similar to FORDISC (version 3.1; Forensic Anthropology Center, University of Tennessee, Knoxville, TN, USA) [60,61], to enable practical population comparisons in forensic settings. This opens the door to integrating machine learning and computer vision methods in future research, with the potential to enhance sensitivity to subtle shape differences and improve classification performance where traditional statistical approaches may fall short.

## 5. Conclusions

Palatal shape variation covaries across all three dimensions, where changes in one variable are often associated with coordinated changes in others. Geometric morphometric analysis revealed statistically significant differences in palatal shape among the four populations examined, with the exception of the closely related Malay and Chinese groups. The European population were associated with a higher palatal vault, coupled with a narrower and longer palate, a more vertically developed palatal contour, and a more angular palatal arch with pronounced tapering in the anterior region compared to the Malay-Chinese cluster. The Indian population appeared intermediate between the Malay-Chinese cluster and the European group in terms of palatal morphology. This approach is quantifiable, population-informative and useful in a forensic context, bioarchaeological research and studies of human craniofacial variation.

The overall classification accuracy of the model was moderate, showing better performance in identifying European and Indian populations than Chinese and Malay populations. The limited precision and recall caution against relying on palatal shape alone for definitive classification of population affinities. The relatively high specificity suggests, however, some utility of palatal shape in excluding population groups. These findings highlight the potential of 3D palatal shape analysis as a supplementary tool in forensic and anthropological contexts. Nonetheless, its application should be considered alongside other morphological, genetic, or contextual data to improve accuracy in estimating population affinity.

## Figures and Tables

**Table 1:** Metrics used for classification performance.

Metric	Formula	Definition
Sensitivity (Recall)	$\frac{\text{True positive}}{\text{True positive}+\text{False negative}}$	Ability to correctly identify the class
Specificity	$\frac{\text{True negative}}{\text{True negative}+\text{False positive}}$	Ability to correctly exclude other classes
Precision	$\frac{\text{True positive}}{\text{True positive}+\text{False positive}}$	Ability to correctly identify positive instances among all predicted positives.
F1 Score	$2\times \frac{\text{Precision}\times \text{Recall}}{\text{Precision}+\text{Recall}}$	Harmonic mean of precision and recall

**Table 2:** Results of Procrustes ANOVA testing the effects of population, sex, and their interaction on shape variation. The R^2^ values indicate the proportion of total shape variance explained by each factor. A significant main effect of population was observed, along with a smaller but significant effect of sex. No significant interaction effect was found between population and sex. Asterisks (*) indicate statistical significance (*p* < 0.05).

Factor	R^2^	F	*p*-Value	Interpretation
Population	0.12	18.46	<0.01 *	Larger, significant effect
Sex	0.01	3.22	0.01 *	Small but significant effect
Population:Sex interaction	0.01	1.36	0.14	Insignificant, no evidence that the effect of sex differs across populations.

**Table 3:** Eigenvalue and percentage variance explained by the PCs.

Principal Component (PC)	Eigenvalue	% Variance	Cumulative %
1	0.0024	26	26
2	0.0013	14	40
3	0.0009	10	50
4	0.0005	6	56
5	0.0004	5	61

**Table 4:** Shape variation explained by each PC.

PC (% Variance)	Shape Features	Shape Interpretation (±2 SD)
PC1 (26%)	Curvature of palatal vault Transverse contour (lateral slope) of palate Transverse dispersion of palate Palatal arch shape	More pronounced surface curvature (domed) of palatal vault with increased convexity of palatal roof, steeper lateral contours and reduced transverse spread. Narrower, more V-shaped palatal arch.
PC2 (14%)	Left-right asymmetry in lateral slopes of palate Anteroposterior redistribution of palatal vault along sagittal axis	Lateral asymmetry with side-specific expansion. Differential curvature across anterior–posterior region, producing a twist-like deformation on sagittal axis.
PC3 (10%)	Regional anteroposterior curvature of palatal vault along sagittal axis Localised deformation of palatal surface	Posterior palate shows increased convexity while anterior palate is relatively flattened, producing a reciprocal curvature pattern along the sagittal axis. Subtle diagonal surface displacement, producing hinge-like deformation in specific local regions of the palate.
PC4 (6%)	Central vs. peripheral shape variation in palate	Palatal surface shows increased convexity in the central region, with relative flattening of peripheral areas.
PC5 (5%)	Palatal arch curvature at premolar region	Localised curvature changes in palatal arch in the premolar region.

**Table 5:** Eigenvalue and percentage variance explained by the CVs.

Canonical Variate	Eigenvalue (Canonical Root)	% Variance	Cumulative %
CV1	0.41	88%	88%
CV2	0.04	9%	97%
CV3	0.01	3%	100%

**Table 6:** Canonical variate (CV) loadings for the first five PCs.

PC	CV1	CV2	CV3
1	−18.12	−12.91	−1.68
2	−6.68	6.22	4.99
3	7.93	3.68	−10.58
4	27.03	−28.76	−15.17
5	14.73	−16.40	40.86

**Table 7:** Pairwise Mahalanobis distance.

	Australian European	Malaysian Malay	Malaysian Chinese	Malaysian Indian
Australian European	—	—	—	—
Malaysian Malay	1.55 (*p* < 0.01)	—	—	—
Malaysian Chinese	1.61 (*p* < 0.01)	0.47 (*p* = 0.10)	—	—
Malaysian Indian	1.15 (*p* < 0.01)	0.72 (*p* < 0.01)	0.60 (*p* < 0.01)	—

**Table 8:** Confusion matrices showing (A) the original classification, (B) cross-validated classification results from Canonical Variate Analysis and (C) classification using the testing datasets. Classification performance of the model is presented in (D).

**(A) Original classification. Accuracy = 49%.**					
	**Predicted Group**	**Chinese**	**Indian**	**Malay**	**European**
**Actual Group**					
Chinese		44%	14%	24%	17%
Indian		17%	40%	22%	21%
Malay		23%	17%	47%	13%
European		5%	16%	14%	64%
**(B) Cross-validated classification. Accuracy = 44%**					
Chinese		41%	17%	26%	17%
Indian		19%	33%	24%	23%
Malay		30	19%	38%	13%
European		7%	17%	13%	63%
**(C) Classification in the independent test dataset. Accuracy = 45%**					
Chinese		40%	10%	50%	0%
Indian		10%	50%	20%	20%
Malay		40%	20%	30%	10%
European		10%	10%	20%	60%
**(D) Classification performance in the independent test dataset.**					
**Group**		**Sensitivity (Recall)**	**Specificity**	**Precision**	**F1 Score**
Chinese		0.40	0.80	0.40	0.40
Indian		0.50	0.87	0.56	0.53
Malay		0.30	0.70	0.25	0.27
European		0.60	0.90	0.67	0.63

## Data Availability

The data that support the findings of this study are available from the corresponding author upon reasonable request.
